# A systematic overview of prospective cohort studies of cardiovascular disease in sub-Saharan Africa: reply to Bovet *et al.*, and Gao *et al.*

**Published:** 2012-09

**Authors:** Andre Pascal Kengne, Bongani M Mayosi

**Affiliations:** National Collaborative Research Programme on Cardiovascular and Metabolic Diseases, South African Medical Research Council, Cape Town and Department of Medicine, Groote Schuur Hospital and University of Cape Town, Cape Town, South Africa; Department of Medicine, Groote Schuur Hospital and University of Cape Town, Cape Town, South Africa

## Dear Sir

Two groups of investigators have recently provided evidence supporting the need for elaborated longitudinal studies to inform successful health service and policy solutions to the growing problem of chronic and cardiovascular disease in sub-Saharan Africa (SSA).^1^-^3^ In one of those studies, published in the *Cardiovascular Journal of Africa*,^1^ our group reached such a conclusion on the basis of a systematic review of relevant existing cohort studies conducted in SSA, published and indexed to MEDLINE from 1966 to October 2009.^1^

The feedback received from colleagues both from Africa and beyond testifies to the interest and also the expectations of the scientific community at large for longitudinal studies on chronic diseases in SSA. We are particularly grateful to Drs Bovet and Shamlaye,^4^ and Drs Gao and Yuan,^5^ who through two letters published in the *Cardiovascular Journal of Africa*, have made a significant contribution to the debate.

Drs Bovet and Shamlaye^4^ provided evidence suggesting that our review missed some relevant studies fulfilling our entry criteria and published in leading medical journals. They further suggested that we omitted some SSA countries from our search. We did acknowledge in the limitations sections of our article that for a number of reasons, there was still a possibility that our search did not capture all relevant studies. Therefore, we welcome the contribution of Bovet and Shamlaye and call for an ongoing register of African cohort studies, possibly in the columns of the *Cardiovascular Journal of Africa* along the lines of the cohort profiles in the *International Journal of Epidemiology*.^6^

However, of the eight studies listed by the two colleagues, at least four do not fulfil the eligibility criteria of our review, including a study from Mauritius published one year after the completion of our review,^7^ a study with a follow-up duration shorter than six months,^8^ one in which none of the predictors of interest was assessed at baseline,^9^ and one cross-sectional study with no follow-up component.^10^ It would have been more appropriate to repeat the systematic search using our strategy, or any other judged appropriate by the authors, and quantify the gap, if any, between our study and what should have been optimal.

Furthermore, unlike the authors’ suggestion, we made no restriction by country or importance of the journal of publication in our search, nor did we claim that cohort studies have not been conducted in Africa. Notwithstanding the above shortcomings, the many similarities between the studies presented by the authors and those included in our review in terms of limitations of the data available further strengthen our conclusions. Some of those limitations include the small sample size, the short duration of follow up and the high rate of drop-out during follow up.

Drs Gao and Yuan also suggested that our work did not cover all aspects of the relationship between cardiovascular disease and related risk factors.^5^ Their claim is absolutely right and would apply to even the landmark Framingham study,^11^,^12^ which over the course of more than 60 years, has not yet covered all aspects of the interaction between determinants and cardiovascular diseases. The broadness of the cardiovascular disease field definitely invited some prioritisation in the course of our study. This prioritisation was based on the knowledge from the literature of important cardiovascular diseases and their major determinants, those cardiovascular diseases and risk factors which are likely more important in the African setting.

From our experience researching cardiovascular diseases in Africa, we had several strong indicators that existing relevant cohort studies, if any, would singly not be sufficient to address major gaps in knowledge. Therefore our aim, as stated in our article, was to identify existing cohort studies and assess whether these could be combined to increase the statistical power for answering major research questions, particularly through individual participant data meta-analyses, as done in the Asia–Pacific region over the last decade, for instance.^13^

For such a purpose, targeting major cardiovascular diseases and risk factors seems in our opinion to be an appropriate approach and would ultimately capture the studies with relevance for the investigation of other risk factors. In the absence of individual participant data to quantify and compare the contribution of risk factors to disease occurrence, we are unable to understand what sort of classification of risk factors the authors are referring to, which incidentally, was not an aim of our study.

The time has come for the establishment of a prospective register of African cohort studies on cardiovascular and other chronic diseases in order to ensure the dissemination of valuable knowledge, the identification of research needs, and the promotion of health in the African region.
